# Impact of Venous Drainage on Tumor Lateralization in Inferior Petrosal Sinus Sampling: Reappraisal With Literature Review

**DOI:** 10.7759/cureus.95215

**Published:** 2025-10-23

**Authors:** Atsushi Ishida, Noriaki Tanabe, Naoko Inoshita, Koji Takano, Hideki Shiramizu, Haruko Yoshimoto, Go Matsuoka, Shozo Yamada

**Affiliations:** 1 Hypothalamic and Pituitary Center, Moriyama Memorial Hospital, Tokyo, JPN; 2 Pathology Department, Moriyama Memorial Hospital, Tokyo, JPN; 3 Endocrinology Department, Moriyama Memorial Hospital, Tokyo, JPN

**Keywords:** cushing’s disease, inferior petrosal sinus sampling, prolactin, tumor lateralization, venous drainage

## Abstract

Introduction

Venous drainage patterns may compromise the ability of inferior petrosal sinus sampling (IPSS) to correctly lateralize corticotroph tumors in Cushing’s disease. We aimed to reappraise this effect in patients proceeding to transsphenoidal surgery (TSS) and to contextualize our findings with a brief review of the literature.

Methods

We retrospectively reviewed the records of 37 consecutive patients who underwent IPSS at our institution between April 2018 and March 2025. Of these, 28 subsequently underwent TSS based on IPSS findings and other clinical data. Corticotroph pituitary neuroendocrine tumors were histologically confirmed in 25 patients. We compared IPSS-indicated lateralization with the intraoperatively determined and histopathologically confirmed tumor side, and we systematically evaluated the influence of venous drainage patterns on false lateralization.

Results

Of the 25 histologically confirmed cases, IPSS lateralization agreed with the actual tumor side in 22 patients (88%), including eight with venous drainage from the tumor side toward the contralateral side. In contrast, three patients (12%) showed false lateralization, in which IPSS indicated the opposite side of the actual tumor. All three false-lateralized cases exhibited dominant contralateral venous drainage, and prolactin-adjusted values did not alter the interpretation. Overall, among 11 patients with dominant contralateral drainage, IPSS misidentified the tumor side in three (27%).

Discussion

IPSS demonstrated high lateralization accuracy in our cohort; however, dominant venous drainage from the tumor to the contralateral side may result in false lateralization. Careful assessment of venous outflow patterns is essential, particularly when IPSS findings contradict MRI-based lateralization, to support appropriate surgical decision-making.

## Introduction

Inferior petrosal sinus sampling (IPSS) is a well-established diagnostic tool for confirming Cushing’s disease (CD) and lateralizing adrenocorticotropic hormone (ACTH)-secreting pituitary neuroendocrine tumors (PitNETs) when magnetic resonance imaging (MRI) results are inconclusive or equivocal [1-3]. IPSS remains highly accurate for distinguishing pituitary from ectopic ACTH secretion, with pooled sensitivity around 94-96% and specificity around 90-100%, as confirmed in a recent meta-analysis [4]. While IPSS offers high sensitivity and specificity for diagnosing ACTH-dependent hypercortisolism, its accuracy in lateralizing tumors within the pituitary gland remains variable. Several studies have reported concordance rates between IPSS lateralization and surgical or pathological tumor location, ranging from 50% to 70%, raising concerns about its reliability for surgical guidance [4-6]. One potential factor influencing the accuracy of IPSS lateralization is the pattern of venous drainage from the pituitary gland [5-7]. Venous drainage patterns and lateralization associated with IPSS have been extensively investigated and reported from the 1990s to the early 2000s [8-10]. With recent advancements in diagnostic modalities for CD, including improved MRI techniques [11,12] and the introduction of positron emission tomography (PET) imaging [13,14], the relative importance of IPSS in the diagnostic algorithms has arguably declined. However, it may be worthwhile to revisit the issues of lateralization and venous drainage in IPSS in contemporary CD cases that remain diagnostically challenging. Thus, IPSS is an infrequently performed procedure that requires a high level of technical expertise. One of the challenges in this field is the difficulty in accumulating a large number of cases with homogeneous data. Additionally, measuring IPS prolactin (PRL) levels has been proposed to enhance the diagnostic accuracy of tumor laterality [15,16]. However, recent studies indicated that PRL measurements do not independently contribute to the diagnosis or lateralization of tumors [17,18]. In this retrospective single-center study, we reassessed the impact of venous drainage patterns on the accuracy of IPSS lateralization in patients with MRI-negative or equivocal CD. Our primary outcome was the concordance between IPSS-predicted lateralization (with and without PRL correction) and the actual tumor location confirmed intraoperatively and histopathologically. As a secondary outcome, we evaluated the relationship between venous drainage patterns and false lateralization. By integrating these findings with a literature review, we aimed to clarify the mechanisms underlying discordant IPSS results and provide practical guidance for interpreting IPSS in surgical planning.

## Materials and methods

Target patients

We conducted a retrospective review of all patients in our hospital between April 2018 and March 2025 who underwent IPSS for presumed ACTH-secreting PitNETs with negative or equivocal imaging findings. All the patients were referred from institutions throughout Japan. All patients underwent a spoiled gradient echo sequence (SPGR) MR study (3.0T Ingenia, Philips Healthcare, Best, Netherlands) at our hospital, and the results were independently reviewed by S.Y. and A.I. In cases in which both clinicians suspected the presence of a tumor but remained uncertain about the results, IPSS was performed after obtaining informed consent from the patient. Corticotroph PitNETs were confirmed by reviewing the final histopathological diagnosis from the intraoperative specimen, along with the operative notes. Patients with radiologically evident tumors, a history of prior pituitary surgery or radiation, incomplete IPSS data, or missing follow-up records were excluded. Informed consent for the use of the patient’s data was obtained by an opt-out approach in all included patients. This clinical research was approved by the Clinical Research Ethics Committee of our hospital.

IPSS procedure

**Figure 1:**
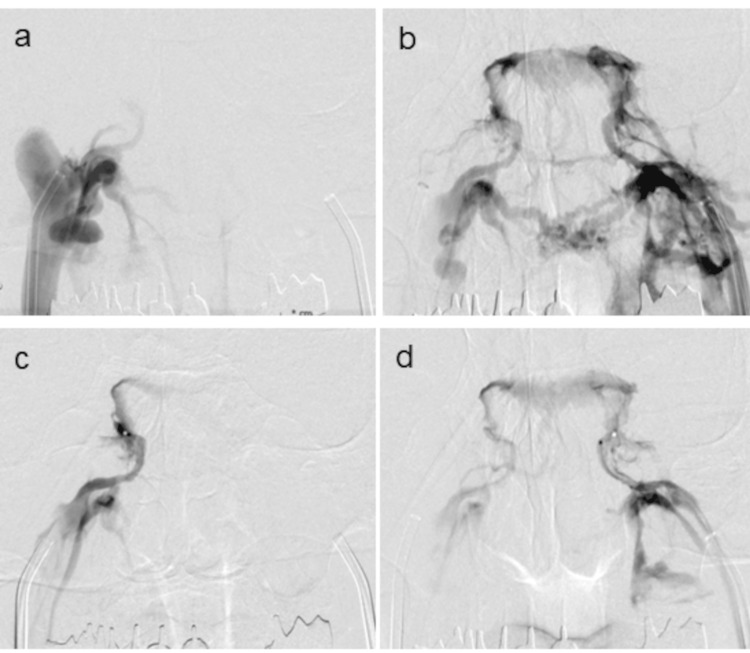
Routine venography for assessing the direction of bilateral cavernous sinus drainage. Venography was performed using a 6-Fr guiding catheter positioned at the exit of the IPS (a, b) and a microcatheter placed within the IPS (c, d). Venography from the right side (a, c) demonstrated significantly weaker opacification compared to that from the left side (b, d), indicating dominant venous drainage from the left to the right side. IPS: inferior petrosal sinus

All the IPSS procedures were performed by a neuro-endovascular specialist (A.I.) in a standardized manner. Access to the right and left common femoral veins was obtained, and a 6-Fr vascular access sheath on the right side and a 7-Fr sheath on the left side were inserted, allowing peripheral blood samples to be drawn from the oversized sheath during the procedure. Using a standard angiographic technique, a 6-Fr guide catheter was advanced into both internal jugular veins (IJVs), and venography was subsequently performed (Figures 1a-1b). A 0.027-inch microcatheter was navigated bilaterally through the guide catheter to the distal IPS. Venography was performed using a microcatheter placed in the IPS to confirm the direction of venous crossflow (Figures 1c-1d). Blood samples from the IJV and IPS were then collected from the guide catheters and microcatheters, respectively. As part of the IPSS protocol, ACTH levels from the right and left IPSs were documented at baseline and at two, five, and 10 minutes after stimulation with human corticotropin-releasing hormone (CRH) [19]. Peripheral ACTH levels at these time points were also obtained as controls for IPS measurements. At each point of ACTH sampling, blood was simultaneously drawn from the bilateral IPSs and peripheral blood for PRL measurement. There were no complications of IPSS, as all procedures were performed with meticulous care.

Data interpretation

A central-to-peripheral (C/P) ACTH gradient of ≥2.0 at baseline or of ≥3.0 after CRH administration suggested a pituitary source for ACTH [20]. If these criteria are satisfied, then no additional calculations are required. If the extent of ACTH elevation did not meet these criteria, we followed an algorithm that has been used for a long time [21] and concurrently measured PRL concentrations to verify proper sampling. The C/P PRL ratios were calculated at each time point to confirm proper IPS venous sampling, as defined by a C/P PRL ratio >1.8. Localization results from the ACTH ratio and PRL-adjusted ACTH ratio were compared with surgical findings and pathology. An intersinus gradient was defined as the measured mean ACTH concentration of the central left/right IPS divided by the mean ACTH concentration of the central right/left IPS; a value ≥1.4 was indicative of lateralization [22].

Surgery

Patients with bilateral IPSS results consistent with CD underwent endoscopic TSS as previously described [23]. The pituitary gland was accessed via a horizontal approach extending to both cavernous sinuses (CSs). If no tumor was visible, the side indicated by IPSS was first explored by dividing the gland vertically. If still negative, the contralateral side was similarly explored. When no tumor was found, horizontal transection followed by systematic vertical sectioning of the anterior lobe into 1-2 mm slices was performed to detect occult lesions.

Histopathological analysis

The definitive diagnosis of CD was confirmed by histopathological examination. Histopathological analysis was performed by an experienced neuropathologist (N.I.). Specimens were evaluated using hematoxylin and reticulin staining as well as immunohistochemistry for ACTH and other pituitary hormones.

Statistical analysis

Because the sample size was limited, no formal hypothesis testing was performed. Data are presented descriptively as absolute numbers, percentages, and ranges to allow transparent interpretation and reproducibility.

## Results

Patient demographics and analysis overview

**Figure 2:**
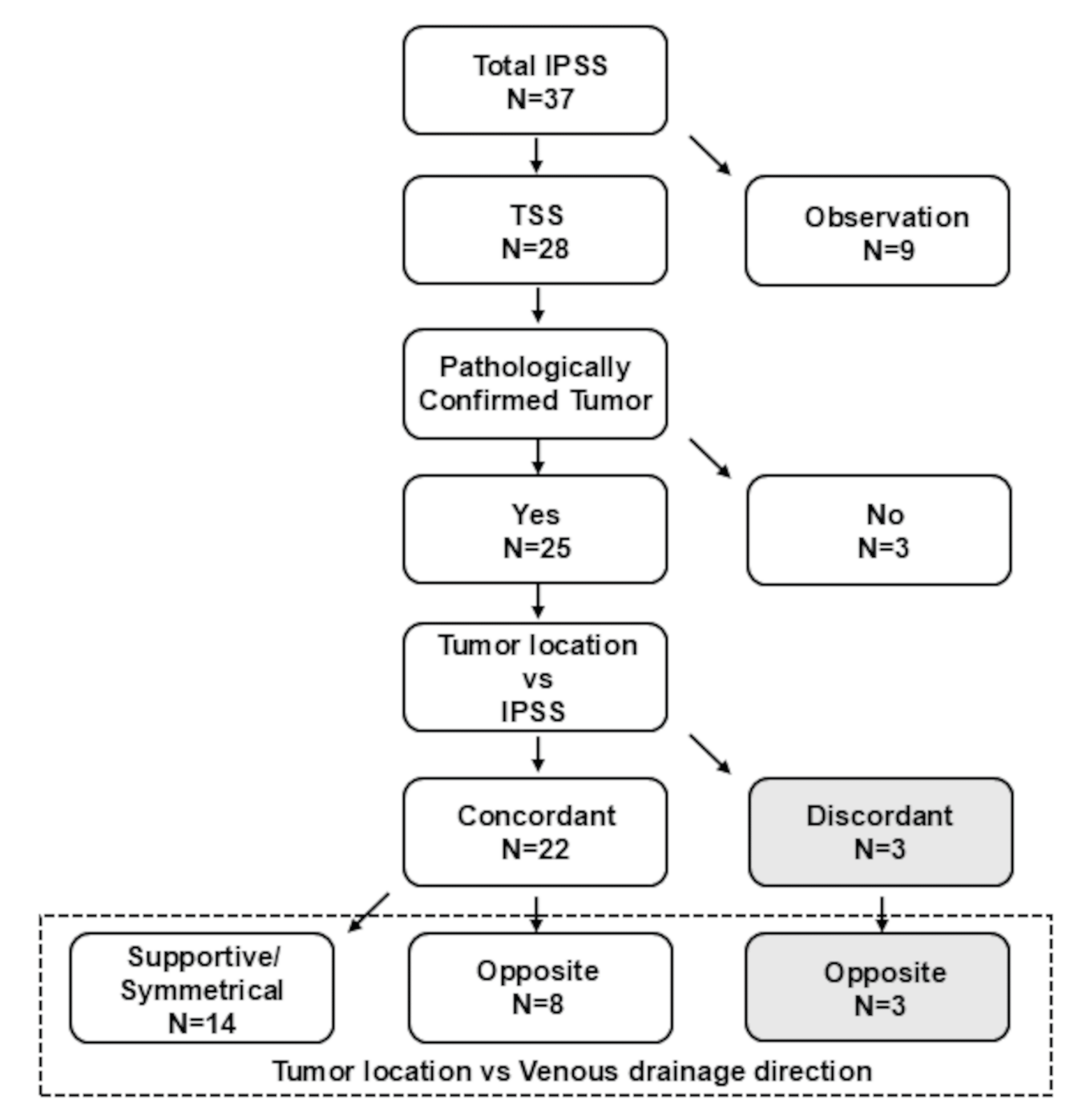
Flow diagram of patient inclusion for the present study focusing on tumor location vs. venous drainage direction. IPSS: inferior petrosal sinus sampling, TSS: transsphenoidal surgery

Because there was no comparison between treatment groups and the sample size was relatively small, the demographic information is presented here descriptively rather than in tabular form. All 37 cases were analyzed, and the findings are summarized in Figure 2 and in the following text. Surgical outcomes, including concordant and discordant cases, are described in detail to provide a comprehensive understanding of the cohort without formal statistical hypothesis testing.

IPSS findings

Thirty-seven patients (29 women and eight men) with equivocal MRI findings underwent the IPSS to determine the origin of ACTH hypersecretion using a C/P ACTH gradient (Figure 2). The mean age of the 37 patients was 42.5 years (range: 22-80 years). Successful bilateral IPS cannulation was achieved via the ipsilateral IJV in all patients. Four patients did not show ACTH C/P ratios greater than the cut-off values of ≥2 before and ≥3 after CRH administration. In cases where ACTH levels in the IPS were clearly low or where peripheral ACTH levels were extremely low, raising concerns about cyclic or pseudo-Cushing’s syndrome, we ultimately concluded that a central source could not be reliably determined even if conventional diagnostic criteria were met. As a result, TSS was not performed in nine cases (five with insufficient ACTH gradients and four clinically considered pseudo-/cyclic Cushing’s syndrome).

Surgical outcomes

In the remaining 28 cases, in which the results of IPSS strongly suggested a central origin and considering the physical findings and detailed endocrinological evaluations indicating a high likelihood of central etiology, TSS was performed after obtaining informed consent. Pathologically confirmed ACTH-producing tumors were identified in 25 patients. Despite employing the extensive procedures described in the Methods section, we were unable to identify and localize the tumor in the three cases (Figure 2). Of these 25 cases, the tumor lateralization identified by IPSS was concordant with the actual tumor location in 22 cases and discordant in three cases. In all three discordant cases, strong venous flow from the tumor side to the contralateral side was observed (Figure 2). Among the 22 concordant cases, venous flow from the tumor side to the contralateral side was noted in eight cases (Figure 2). In these cases, the direction of the venous flow did not affect the interpretation of tumor lateralization. Therefore, in cases where venous drainage from the tumor side to the contralateral side was observed, the IPSS lateralization result was opposite to the actual tumor location in three of the 11 cases.

Representative cases: three cases of IPSS-tumor side discordance

Case 1: 33-Year-Old Woman

**Figure 3:**
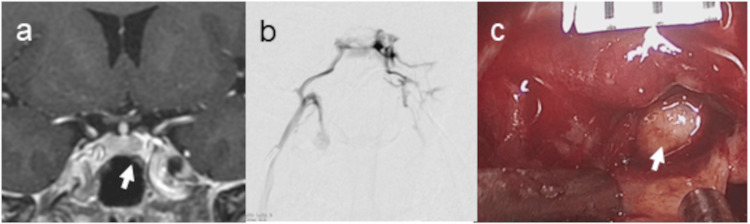
Case 1. A patient in whom venous drainage led to false-lateralization on IPSS, which was not corrected by prolactin adjustment (Table 1). A 33-year-old woman. An SPGR MR image shows equivocal lesions on the left side (arrow) (a). Venography from the left IPS demonstrated dominant venous drainage from the left to the right side (b). Intraoperative image demonstrates tumor located in the left lateral wing (arrow) (c). IPSS: inferior petrosal sinus sampling, SPGR: spoiled gradient echo sequence

IPSS revealed a markedly elevated right-to-left IPS ACTH ratio, strongly suggesting right-sided tumor localization. Notably, the ratio exceeded 20-fold at five minutes after CRH stimulation (Table 1). Adjustment based on PRL values did not alter this interpretation, as all right-to-left ACTH ratios remained significantly elevated (Table 1). However, SPGR MRI demonstrated an equivocal lesion on the left side, with findings suggestive, but not definitive, of a left-sided tumor (Figure 3a). Furthermore, selective venography from the left IPS showed dominant parasellar venous drainage from the left to the right side (Figure 3b), raising the possibility that lateralization by IPSS may have been influenced by the venous drainage pattern. Given the inconclusive but suggestive MRI findings and venographic evidence of left-to-right venous flow, surgical exploration was initiated on the left side. Intraoperative inspection revealed a tumor within the lateral wing of the left pituitary gland (Figure 3c), which was subsequently resected and histopathologically confirmed to be an ACTH-secreting tumor. The patient achieved complete postoperative remission.

**Table 1:** IPSS results of Case 1. Rt. IPS/Lt. IPS ratio of ACTH strongly indicated right-sided tumor localization (gray shaded). PRL-adjusted value did not alter the interpretation (gray shaded). IPSS: inferior petrosal sinus sampling, ACTH: adrenocorticotropic hormone, PRL: prolactin

	ACTH (pg/mL)			ACTH ratio	PRL (ng/mL)			ACTH/PRL ratio		
Time after CRH	P	Rt. IPS	Lt. IPS	Rt. IPS/Lt. IPS	P	Rt. IPS	Lt. IPS	Rt.	Lt.	Rt./Lt.
Zero minutes	19.3	319	115	2.8	16.4	103	84.7	3.1	1.4	2.3
Two minutes	19.4	7720	393	19.6	16.3	203	79.4	38	5	7.7
Five minutes	55.5	4810	236	20.4	17.1	170	56.5	28.3	4.2	6.8
10 minutes	16.4	989	65.1	15.2	17.6	77.4	30.9	12.8	2.1	6.1

Case 2: 40-Year-Old Woman

**Figure 4:**
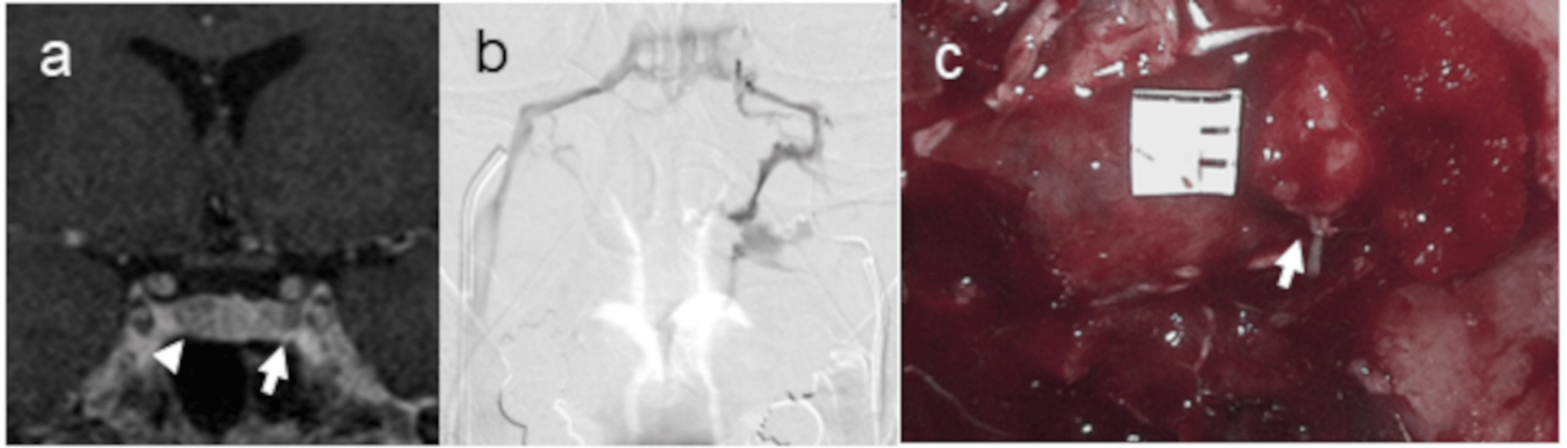
Case 2. A patient in whom venous drainage led to false-lateralization on IPSS, which was not corrected by prolactin adjustment (Table 2). A 40-year-old woman. An SPGR MR image shows equivocal lesions on the left side (arrow) as well as right side (arrowhead) (a). Venography from the left IPS demonstrated dominant venous drainage from the left to the right side (b). Intraoperative image demonstrates tumor located in the left lateral wing (arrow) (c). IPSS: inferior petrosal sinus sampling, SPGR: spoiled gradient echo sequence

IPSS revealed marked lateralization of ACTH secretion, favoring the right side (Table 2). Although baseline ACTH concentrations were marginally higher in the left IPS before CRH administration, this distribution shifted notably after stimulation. By 10 minutes post-CRH, ACTH levels in the right IPS exceeded those in the left IPS by more than 11-fold. A comparable temporal shift was observed in the PRL-adjusted ACTH values; while the initial and two-minute values were slightly higher on the left, the five- and 10-minute measurements demonstrated a reversal, with a pronounced increase on the right. This pattern confirmed that the PRL correction did not affect the interpretation of lateralization. SPGR MRI demonstrated equivocal findings with suspicious lesions detected on both sides of the gland (Figure 4a). Venographic assessment revealed a dominant venous flow from the left toward the right IPS (Figure 4b). Given the ambiguous imaging findings and strong right-sided IPSS, surgical exploration was initiated on the right side. No lesions were detected on that side. Subsequent exploration of the left side revealed a tumor (Figure 4c), which was confirmed by histopathological examination. Endocrinological remission was achieved.

**Table 2:** IPSS results of Case 2. Rt. IPS/Lt. IPS ratio of ACTH strongly indicated right-sided tumor localization (gray shaded). PRL-adjusted value was still controversial (gray shaded). ACTH: adrenocorticotropic hormone, IPSS: inferior petrosal sinus sampling, PRL: prolactin, SPGR: spoiled gradient echo sequence

	ACTH (pg/mL)			ACTH ratio	PRL (ng/mL)			ACTH/PRL ratio		
Time after CRH	P	Rt. IPS	Lt. IPS	Rt. IPS / Lt. IPS	P	Rt. IPS	Lt. IPS	Rt.	Lt.	Rt./ Lt.
Zero minutes	69.8	2340	4820	0.46	19.8	116	38.8	20.2	124.2	0.16
Two minutes	75.4	28300	28700	0.99	14.3	176	44.6	160.8	643.5	0.25
Five minutes	341	367000	55500	6.6	14.2	948	188	387.1	295.2	1.3
10 minutes	709	143000	12800	11.2	15	99	33.6	1444	381	3.8

Case 3: 50-Year-Old Man

**Figure 5:**
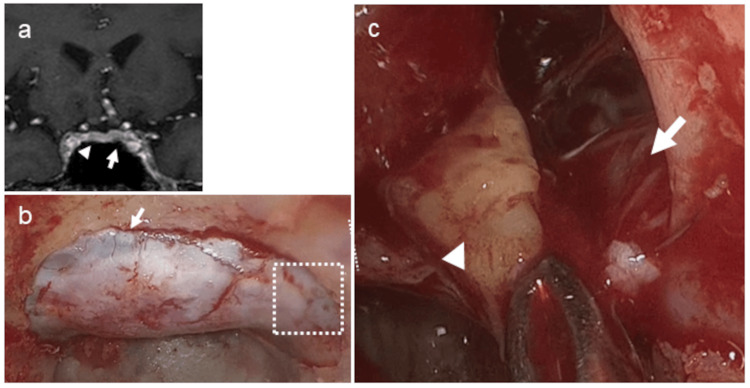
Case 3. A patient in whom markedly diminished drainage through the ipsilateral cavernous sinus affected the interpretation of lateralization, as reflected in the prolactin measurements (Table 3). A 50-year-old man. An SPGR MR image shows equivocal lesions on the left side (arrow) as well as the right side (arrowhead) (a). Intraoperative images are shown in (b) and (c). The sellar floor was widely exposed, revealing a remarkably developed intercavernous sinus (arrow) (b). The area within the dotted square in (b) after opening the dura is magnified in (c), showing tumor removal (arrowhead). The left internal carotid artery was exposed (arrow), and the left CS was widely opened. Notably, there was almost no venous bleeding, allowing clear visualization of the anatomy within the left CS without suction. ACTH: adrenocorticotropic hormone, CS: cavernous sinus, IPSS: inferior petrosal sinus sampling, P: peripheral, PRL: prolactin, SPGR: spoiled gradient echo sequence

IPSS demonstrated a markedly elevated ACTH ratio between the right and left IPS, strongly indicating right-sided tumor localization. This lateralization was already evident prior to CRH stimulation, but became even more pronounced following stimulation, with the right-to-left ACTH ratio reaching an extraordinary 100-fold at 10 minutes post-stimulation (Table 3). Despite the technically successful catheterization of the left IPS using a microcatheter in which the catheter tip was adequately advanced into the distal segment and blood samples were obtained without any procedural difficulty, the PRL ratio between the left IPS and peripheral blood remained persistently below 1.8 (Table 3). Such a low PRL gradient raises concerns regarding the reliability and representativeness of hormone concentrations obtained from the left IPS, suggesting possible underestimation of hormone levels due to suboptimal venous drainage. Coronal SPGR MRI revealed equivocal findings with subtle signal alterations on both the left and right sides of the pituitary gland and no clear radiographic lateralization (Figure 5a). Further insight was obtained through venographic assessment during IPSS, indicating dominant venous drainage from the left to the right side (data not shown). The intraoperative findings corroborated the venous anatomy (Figures 5b-5c). The sellar floor was opened extensively, exposing the prominently developed intercavernous sinus (Figure 5b). A magnified view of the boxed area in Figure 5b (Figure 5c) shows the left CS after surgical opening, illustrating the resection of the tumor with the medial wall of the CS, which had been invaded by the tumor. The left internal carotid artery was clearly visualized, and the left CS was widely exposed. Remarkably, venous bleeding was minimal throughout the procedure, permitting an unimpeded view of the anatomical structures within the left CS without suction (Figure 5c). Although such a phenomenon is rather rare, it has been postulated that the left CS contributes minimally to normal venous outflow. Instead, venous drainage from the left side appeared to occur predominantly through the intercavernous sinus on the contralateral side. This aberrant drainage pattern likely results in the dilution or diversion of hormone concentrations, including PRL, thereby leading to falsely low values from left-sided sampling and potentially misleading interpretation of lateralization during IPSS. Endocrinological remission was also achieved.

**Table 3:** IPSS results of Case 3. Rt. IPS/Lt. IPS ratio of ACTH strongly indicated right-sided tumor localization (gray shaded). Lt. IPS/ P ratios of PRL were less than 1.8 (gray shaded). ACTH: adrenocorticotropic hormone, IPSS: inferior petrosal sinus sampling, P: peripheral, PRL: prolactin

	ACTH (pg/mL)			ACTH ratio	PRL (ng/mL)			PRL ratio	
Time after CRH	P	Rt. IPS	Lt. IPS	Rt. IPS/Lt. IPS	P	Rt. IPS	Lt. IPS	Rt. IPS/P	Lt. IPS/P
Zero minutes	32.4	842	26.9	31.3	9.8	101	9.6	10.3	0.9
Two minutes	30.9	1042	25.9	40.2	9.7	131	9.8	13.5	1
Five minutes	28.7	1618	28.7	56.4	9.8	141	9.8	14.4	1
10 minutes	32.8	3360	34.3	98	10	175	10	17.5	1

## Discussion

Although the necessity of IPSS has undoubtedly declined in recent years owing to advances in high-resolution MRI and the emergence of nuclear medicine imaging modalities, such as methionine PET, it remains essential in selected cases where these imaging techniques fail to localize the lesion. When TSS is performed based on IPSS findings, the surgical strategy is often guided by the lateralization indicated by the results. If the lateralization suggested by IPSS is discordant with the actual tumor location, the discrepancy can significantly affect surgical outcomes. The influence of venous drainage patterns on IPSS lateralization has been extensively discussed in the literature from the 1990s to the early 2000s. Although such discussions have become less common in the era of advanced MRI, a few recent studies have provided intriguing insights. We conducted a thorough review of the literature focusing on the impact of venous drainage on IPSS lateralization, and the results are summarized in Table 4. Here, we discuss their findings in comparison with our most recent results.

**Table 4:** A summary of the literature discussing the identification rate of tumor lateralization in relation to venous drainage patterns during sampling procedures. CS, cavernous sinus; CSS, CS sampling; DDAVP, 1-deamino-8-D-arginine vasopressin; IPS, inferior petrosal sinus; IPSS, IPS sampling; oCRH, ovine corticotropin-releasing hormone; VD, venous drainage

First author, year	Number of cases	Sampling location	Secretagogue	Definition of left-right difference in VD pattern	Venous drainage pattern	Correct lateralization of tumor with IPSS
Mamelak et al., 1996 [9]	23	Both IPS and CS	None	Symmetric: contrast injected unilaterally filled IPS with primarily ipsilateral drainage, or if bilateral cross-filling occurred equally. Other patterns were considered asymmetric.	Symmetric 14/23 (61%)	Symmetric drainage: 86% by IPSS, 86% by CSS.
					Asymmetric 9/23 (39%)	Asymmetric drainage: 44% by IPSS, 42% by CSS.
Graham et al., 1999 [10]	59	CS	oCRH	Flow symmetry was determined by rating the degree of opacification of IPS and jugular vein on each side after contrast injection.	Symmetric 46/59 (78%)	Symmetric drainage 89%
					Asymmetric 13/59 (22%)	Asymmetric drainage: 69%
Lefournier et al., 2003 [8]	49	Both IPS and CS	oCRH	By rating the degree of opacification of the IPS on each side after contrast injection. Three positions of the catheters were used.	Symmetric 37/49 (75%)	Symmetric drainage 86%
					Asymmetric 12/49 (25%)	Regardless of VD 57%
					-	CSS did not improve laterality.
Andereggen et al., 2019 [27]	38	IPS	oCRH	Asymmetric: different left-right anatomical venous outflow patterns. Symmetric: no left-right difference	Asymmetric 11 (39%)	Symmetric drainage:100%
					(anatomic symmetry)	Asymmetric drainage: 93%
Ghorbani et al., 2020 [29]	17	IPS	DDAVP	Asymmetric: Contrast from Ipsilateral IPS drains to contralateral IPS; contralateral IPS retains own contrast (dominant side). Symmetric: Both sides drain to contralateral IPS.	Symmetric 4/17 (23.5%)	Symmetric drainage: 50%
					Asymmetric 13/17 (76.5%)	Asymmetric drainage:15%
					-	Parasellar VD laterality utterly matched IPSS lateralization.
Martini et al., 2024 [30]	41	IPS	oCRH	Venography from IPS of tumor side.	-	-
				Cross-filling to contralateral IPS: extensive	Extensive cross-filling 28/41 (68%) (Asymmetric)	Extensive cross-filling: 69%
				No cross-filling to contralateral IPS: minimal	Minimal cross-filling 13/41 (32%)	Minimal cross-filling: 86%

Among these studies, the methods used to evaluate venous drainage asymmetry vary. In many cases, the drainage patterns are categorized as either symmetric or asymmetric. Mamelak et al. were the first to report these patterns in detail [9]. In their study, symmetric venous drainage was observed in 61% of cases, whereas asymmetric drainage was noted in 39% of cases. They investigated the impact of these patterns on the accuracy of lateralization based on IPSS and CS sampling (CSS) separately. In cases with symmetric drainage, both IPSS and CSS demonstrated a high lateralization accuracy of 86%. In contrast, in cases with asymmetric drainage, the accuracy decreased to 42% for both IPSS and CSS. Thus, they concluded that the accuracy of lateralization was comparable when sampling was performed using the IPS or CS. Their report is of particular importance as it presents detailed case-by-case data, including surgical methods and postoperative outcomes. However, the limitation of this study is the absence of stimulation with CRH or 1-deamino-8-D-arginine vasopressin (DDAVP), which are now routinely used. Kaltsas et al. [24] observed an increase in lateralization accuracy from 74% to 83% following CRH administration. Similarly, Doppman et al. [25] reported an improvement from 60% to 73%, whereas Oliverio et al. [26] noted a marked increase from 60% to 94% using CRH.

Similar findings were reported by Graham et al. [10] and Lefournier et al. [8] in studies with larger patient cohorts. Graham et al. analyzed 59 patients who underwent CSS with CRH stimulation and found symmetric venous drainage in 78% of the cases and asymmetric drainage in 22% [10]. The lateralization accuracy was 89% for patients with symmetric drainage and 69% for those with asymmetric drainage. In contrast, Lefournier et al. reported 49 patients who underwent CSS and IPSS after CRH stimulation [8]. They observed symmetric drainage in 79% and asymmetric drainage in 25% of patients, consistent with previous reports. The lateralization accuracy in cases with symmetrical drainage was comparable at 86%. However, the overall lateralization accuracy was 57%, and they concluded that sampling from the CS did not improve lateralization accuracy. Subsequent to these earlier studies, no similar reports had been published.

However, in 2019, Andereggen et al. [27] investigated the asymmetry of venous outflow based on the classification of jugular venous drainage patterns as previously described by Shiu et al. [28]. Their study demonstrated anatomical asymmetry in venous drainage in 39% of the cases. The overall accuracy of lateralization by IPSS was 83% before CRH stimulation and improved markedly to 96% after stimulation. When stratified according to venous symmetry, the accuracy was 100% in anatomically symmetric cases and 93% in asymmetric cases. These findings collectively suggest that symmetric venous outflow patterns are more common than asymmetric ones and that symmetry is associated with higher lateralization accuracy in IPSS, resulting in overall high reliability for lateralization. In contrast, Ghorbani et al. presented findings that directly challenge this conclusion [29]. In their study of 17 cases using DDAVP stimulation, venous outflow asymmetry was observed in 76.5% of the patients. In these cases, the direction of venous drainage appeared to dictate the lateralization outcome entirely, ultimately resulting in markedly low accuracy of tumor localization. Although their findings highlight an important limitation, we believe that this perspective is restrictive and does not reflect real-world clinical practice. Our data indicate that although venous drainage patterns can influence the IPSS results in a subset of cases, the majority of IPSS findings accurately reflect true tumor lateralization, even in the presence of asymmetric drainage. This underscores the continued clinical utility of IPSS for lateralization, provided that venous drainage patterns are carefully assessed and considered in cases where IPSS findings are discordant with MRI findings.

Martini et al. recently published an intriguing study that categorized venographic findings based on the degree of cross-filling from the IPS on the tumor side to that on the contralateral side [30]. Cases with prominent cross-filling were defined as extensive cross-filling and accounted for 68% of the cohort, whereas cases without cross-filling were defined as minimal cross-filling and comprised the remaining 32%. The accuracy of lateralization using IPSS was 69% in the extensive cross-filling group and 86% in the minimal cross-filling group. Interestingly, they also reported that the extensive cross-filling group showed a significantly greater contralateral increase in ACTH levels following CRH stimulation than the minimal cross-filling group. This is the first study to demonstrate a statistically significant association between the degree of cross-filling and the accuracy of lateralization in IPSS. Their analysis was based solely on venography performed on the tumor side, without corresponding imaging from the contralateral side. Therefore, a strict definition of venous drainage asymmetry remains uncertain. However, in cases where deep contrast filling of the contralateral side was observed from the tumor-side injection, as shown in Figure 1, it is reasonable to assume that venography from the contralateral side would show a relatively weak flow in the opposite direction. Thus, the cases classified as “extensive cross-filling” by their criteria likely correspond to asymmetric venous drainage. Conversely, the group defined as “minimal cross-filling” may have included a mixture of cases with truly symmetric drainage and others with dominant contralateral drainage, making it plausible, similar to the observations of Ghorbani et al. [29], that asymmetric cases are in fact more prevalent than previously reported. This contrasts with studies from the late 1990s to the early 2000s, in which symmetric drainage patterns were observed in 60-70% of cases, outnumbering asymmetric cases [8-10]. Martini et al. reported a lateralization accuracy of 69% in patients with extensive cross-filling, a finding that aligns closely with our own result of 73% (eight of 11 cases) [30]. The concordance observed in our eight cases is likely attributable to factors such as a lesser degree of cross-filling and anatomical variations. Conversely, this suggests that in approximately 30% of cases with strong venous drainage from the tumor side to the contralateral side, the IPSS results may demonstrate discordant lateralization.

Several studies have investigated the impact of PRL adjustment on the lateralization accuracy of IPSS. De Sousa et al. reported that all patients with CD had a PRL intersinus gradient towards the tumorous side of the pituitary gland [17]. The mechanism underlying the concomitant sinus ACTH/PRL secretion in corticotroph PitNETs is likely peritumoral PRL production. Contralateral PRL suppression may also occur due to local hyperprolactinemia. Other studies have similarly demonstrated that PRL intersinus gradients favor the ACTH-dominant or tumorous side in CD. In contrast, Mulligan et al. [15] demonstrated that incorporating PRL correction significantly improved lateralization accuracy, increasing the rate from 54% to 75% when a PRL-adjusted intersinus ACTH gradient threshold of >1.4 was applied. Furthermore, when this adjusted gradient was combined with the pituitary MRI findings, the concordance rate for accurate lateralization increased to 82%. In our study, we used a PRL-adjusted ACTH intersinus gradient (Tables 1-2). In both Cases 1 and 2, the PRL-adjusted ACTH ratios showed a slight improvement in lateralization; however, the overall interpretation remained unchanged. In Case 3, the PRL IPS-to-P ratio was below 1.8 on the left side (Table 3), despite successful catheterization into the distal portion of the IPS and technically adequate blood sampling. The intraoperative findings suggested that the left CS contributed minimally to venous drainage, which may account for the unexpectedly low PRL values. These observations underscore the significant impact of venous drainage patterns on the accuracy of lateralization during IPSS. Accordingly, simultaneous PRL measurement and adjustment are essential for improving diagnostic reliability.

In the current landscape where the necessity for IPSS has been diminishing, the complications associated with sampling are becoming increasingly unacceptable. It is essential to obtain the necessary information with minimal invasiveness. In this study, we specifically focused on lateralization accuracy. As discussed previously, venous drainage patterns significantly influence lateralization outcomes, and our findings strongly support this association. When IPSS results contradict suspected tumor laterality based on advanced MRI findings, a comprehensive surgical strategy should be developed that considers PRL levels obtained via simultaneous sampling. Even in cases where the tumor localization was contralateral to the IPSS findings, as reported by Mamelak et al. [9] and supported by our data, thorough bilateral exploration often led to complete tumor resection and clinical remission. However, such favorable outcomes are predominantly observed in high-volume centers with experienced surgeons. In contrast, inadequate exploration may result in failure to identify the lesion or in unintended resection of the normal pituitary tissue. These challenges underscore the importance of tailored operative planning and cautious interpretation of IPSS in facilities with varying expertise.

## Conclusions

This study reappraises the debated impact of venous drainage on IPSS lateralization in CD. Analysis of 25 confirmed cases revealed contralateral drainage as a key cause of false lateralization, and PRL-adjusted gradients did not resolve discordant results. By integrating new data with a literature review, we clarify the mechanisms underlying discordant IPSS findings and provide a more organized perspective on this long-standing issue. These insights emphasize the need for careful consideration of venous drainage patterns when interpreting IPSS results and planning surgery. Future studies with larger multicenter cohorts and standardized venographic assessments are warranted to further validate these findings and to refine criteria for interpreting discordant IPSS results.
